# PD-L1 expression and its correlation with tumor biomarkers in Chinese urothelial bladder cancer

**DOI:** 10.1038/s41598-024-67508-6

**Published:** 2024-07-20

**Authors:** Yu Fan, Tao Dai, Dahong Zhang, Hongqian Guo, Fangjian Zhou, Benkang Shi, Shaogang Wang, Zhigang Ji, Chunxi Wang, Xudong Yao, Qiang Wei, Nanhui Chen, Jinchun Xing, Jinjian Yang, Chuize Kong, Jian Huang, Dingwei Ye, Liqun Zhou

**Affiliations:** 1 Department of Urology, Peking University First Hospital, Beijing, 100034 China; 2 Department of Urology, The Affiliated Cancer Hospital of Xiangya School of Medicine, Central South University and Hunan Cancer Hospital, Changsha, 410006 China; 3 Department of Urology, Zhejiang Provincial People’s Hospital, Hangzhou, 310005 Zhejiang China; 4 Department of Urology, Drum Tower Hospital, Medical School of Nanjing University, Institute of Urology, Nanjing University, Nanjing, 210000 Jiangsu China; 5 Department of Urology, Sun Yat-sen University Cancer Center, Guangzhou, 510060 China; 6 Department of Urology, Qilu Hospital, Cheeloo College of Medicine, Shandong University, Jinan, 250012 China; 7 Department of Urology, Tongji Hospital of Tongji Medical College, Huazhong University of Science and Technology, 1095 Jiafang Avenue, Qiaokou, Wuhan, 430000 Hubei China; 8 Department of Urology, Peking Union Medical College Hospital, Chinese Academy of Science, Beijing, 100005 China; 9 Department of Urology, First Hospital of Jilin University, Changchun, 130000 Jilin China; 10 Department of Urology, Shanghai Tenth People’s Hospital, Tongji University School of Medicine, Shanghai, 200040 China; 11 Department of Urology, Institute of Urology, West China Hospital, Sichuan University, Chengdu, 610041 China; 12 Department of Urology, Meizhou People’s Hospital, Meizhou, 514031 Guangdong China; 13 Department of Urology Surgery, The First Affiliated Hospital of Xiamen University, Xiamen, 361003 Fujian China; 14 Department of Urology, The First Affiliated Hospital of Zhengzhou University, Zhengzhou, 450052 China; 15 Department of Urology, The First Hospital of China Medical University, Shenyang, 110001 China; 16 Department of Urology, Sun Yat-sen Memorial Hospital, Sun Yat-sen University, Guangzhou, 510000 China; 17 Department of Urology, Fudan University Shanghai Cancer Center, Shanghai, 200032 China

**Keywords:** Immune cells, Muscle-invasive urothelial bladder cancer, Prevalence of PD-L1 expression, Tumor biomarkers, Tumor cells, Tumor mutation burden, Cancer, Biomarkers, Oncology, Urology

## Abstract

Data on prevalence of programmed death ligand-1 (PD-L1) expression and its correlation with tumor biomarkers in Chinese patients with muscle-invasive urothelial bladder cancer (MIUBC) are scarce. We investigated the prevalence of PD-L1 expression, PD-L1 expression in tumor cells (TC) and immune cells (IC), and its correlation with tumor biomarkers (CD8+ T cells and tumor mutation burden [TMB]) in Chinese patients with newly diagnosed MIUBC (NCT03433924). Of 248 patients enrolled, 229 with PD-L1 data available were analysed. High PD-L1 expression (≥ 25% of TC or IC with PD-L1 expression) was observed in 120 (52.4%) patients. 59 cases showed positive staining in ≥ 25% of TC, and 82 cases had positive staining in ≥ 25% of IC. High expression of CD8+ T cell and TMB (> 10 mutations/megabase) was observed in 44.5% and 54.1% patients, respectively. A positive correlation was observed between percentage of TC with membrane PD-L1 positivity and CD8+ T cells (0.34; *P* < 0.001) and between IC with membrane PD-L1 positivity and CD8+ T cells (0.44; *P* < 0.001). There is high prevalence of PD-L1 expression in Chinese patients with MIUBC, suggesting that a sizable subset of patients could benefit from immunotherapy. The correlation of PD-L1 expression with tumor biomarkers provide clues for mechanisms underlying the effects of biomarkers for predicting efficacy.

## Introduction

Bladder cancer (BC) is the 10th most common malignant tumor and one of the leading causes of cancer-related deaths globally^1,2^. The global incidence of BC is approximately 573,278 with a 5-year estimated prevalence of 43.8 million worldwide^3,4^. According to the Cancer registry report, China is estimated to have 84,825 new cases of BC and 19,223 BC- related deaths in 2022^5^.

BC is classified as muscle-invasive urothelial bladder cancer (MIUBC) and non-muscle invasive urothelial bladder cancer (NMIUBC) depending upon the tumor infiltration^6^. Among the new cases of BC, around 70% to 80% of cases are diagnosed with NMIUBC^7^, and 15% to 20% of them represent MIUBC^8^. Radical cystectomy with bilateral pelvic lymph node dissection is the gold standard treatment for patients with stage II or III MIUBC^9^; however, 50% of patients with MIUBC still develop metastatic disease within 2 to 3 years of diagnosis^10,11^. Therefore, according to the National Comprehensive Cancer Network (NCCN), chemotherapy is recommended to be used along with surgery in patients with MIUBC^9,12^.

Based on the reports of previous studies, the majority of the evidence for standard guidelines for the diagnosis and treatment of BC in Chinese patients is not originally from China^13^. Therefore, there could be a possibility of inconsistency or discrepancies in treatment strategies in routine clinical practices for BC between China and other countries^13^. Moreover, given the high rate of relapse of patients with MIUBC and that not all patients are suitable for chemotherapy, there has been a pressing need for novel and effective treatment options and strategies for patients globally, particularly in China.

Over the recent years, immune checkpoint inhibitors (ICIs), such as programmed death-1 (PD-1)/programmed cell death ligand-1 (PD-L1) inhibitors, have been studied in different types of cancers, including urothelial cancer, showing robust antitumor activity and a tolerable safety profile^14,15^. For instance, Keynote-052 and Keynote-361 had showed promising effects of pembrolizumab as a first-line therapy in locally advanced and unresectable metastatic, cisplatin-ineligible patients with urothelial cancer^15^. However, evidence to support recommendations in perioperative settings is still lacking^16^. Therefore, to improve the prognosis of patients with MIUBC, perioperative treatment during radical cystectomy is an emerging option, which includes the immediate administration of neoadjuvant or adjuvant ICI^11,17^.

In spite of advances in immunotherapy, identifying patients with MIUBC, who are more likely to respond to ICIs, remains challenging^18^. Over the recent years, PD-L1 expression has emerged as a biomarker to identify such patients^19^. Thus, for an effective response to anti-PD-L1/anti-PD-1 therapies, assessing the status of PD-L1 expression is necessary to modify the treatment strategy^17^. In this regard, the PURE-01 and CheckMate 274 trials were conducted recently, which involved PD-L1 therapy as neoadjuvant and adjuvant therapies, respectively, in patients with MIUBC^20,21^. PD-L1 expression has been shown to be involved in predicting ICI therapy in the PURE-01 and CheckMate 274 trials, whereas other studies, such as DANUBE, did not show any significant correlation between PD-LI expression and survival outcomes. The DANUBE analyzed patients with late-stage disease and used both freshly taken or formalin-fixed, paraffin-embedded (FFPE) tissue tumor samples that were ≤ 3 years old for evaluating PD-L1 expression levels, which could be a possible reason for the nonsignificant correlation between PD-L1 expression and survival outcomes^22^. These results have some contradictions; nevertheless, PD-L1 is still considered the most extensively studied and an efficient biomarker in BC^23^. Further, the use of fresh tumor samples may provide a more accurate assessment of current tumoral PD-L1 expression^24^.

At present, limited information is available about the prevalence of PD-L1 expression in Chinese patients with MIUBC, especially in the perioperative setting. As discussed earlier, the current guidelines for the standard treatment of MIUBC do not have enough data from China, which has encouraged research to fill these knowledge gaps that could aid in making treatment decisions for patients with MIUBC in China. Therefore, we have conducted this prospective, multicenter, epidemiologic study to evaluate the prevalence of PD-L1 expression in Chinese patients with MIUBC, particularly by using fresh tumor samples. The association of PD-L1 expression with tumor immune biomarkers was also investigated.

## Results

### Patient disposition and baseline characteristics

Of 248 patients with MIUBC enrolled in this study, 229 patients with valid PD-LI expression data were included in the analyses. The baseline characteristics of the study cohort are presented in Table 1. The average age of patients was 67.9 years with 108 (47.2%) patients aged ≥ 70 years; the majority of patients (n = 197, 86%) were men. Most of the patients were diagnosed using radical cystectomy (n = 142, 62%) and the most common tumor type was high-grade papillary urothelial cancer, which was found in 221 (96.5%) patients. In terms of the tumor stage, the majority of the patients were diagnosed with stage T2 (n = 129, 57.1%), N0 (n = 160, 70.8%), M0 (n = 188, 83.2%), and AJCC stage II (n = 117, 51.8%) (Table 1).

**Table 1:** Demographic characteristics.

Variable	Total
	(N = 229)
	n (%)
Age, years	
Mean (SD)	67.9 (10.8)
Age group, n (%)	
˂ 70 years	121 (52.8)
≥ 70 years	108 (47.2)
Sex, n (%)	
Female	32 (14.0)
Male	197 (86.0)
BMI, kg/m^2^	
Mean (SD**)**	23.2 (3.1)
Nicotine use, n (%)	
Current	54 (23.6)
Former	31 (13.5)
Never	144 (62.9)
AJCC stage, n (%)	N = 226
Stage II	117 (51.8)
Stage III	86 (38.1)
Stage IV	23 (10.2)
Duration since diagnosis, months	N = 228
Mean (SD)	0.6 (1.9)
Primary tumor stage, n (%)	N = 226
T2	129 (57.1)
T3	58 (25.7)
T4	39 (17.3)
Regional lymph nodes, n (%)	N = 226
N0	160 (70.8)
N1	26 (11.5)
N2	21 (9.3)
N3	4 (1.8)
NX	15 (6.6)
Distant metastases, n (%)	N = 226
M0	188 (83.2)
M1	8 (3.5)
MX	30 (13.3)
Tumor grade, n (%)	
Papillary urothelial neoplasm of low malignant potential	4 (1.7)
Low-grade papillary urothelial carcinoma	4 (1.7)
High-grade papillary urothelial carcinoma	221 (96.5)
Method of determination, n (%)	
Partial cystectomy	4 (1.7)
Radical cystectomy	142 (62.0)
Transurethral resection	78 (34.1)
Biopsy	5 (2.2)

Duration since diagnosis (month) = (date of enrollment – date of diagnosis + 1)/30.

AJCC, The American Joint Committee on Cancer; BMI, body mass index; MIUBC, muscle invasive urothelial bladder cancer; N, number of subjects in the analysis population; n, number of subjects in the specified category; PD-1, programmed cell death protein 1; PD-L1, programmed cell death protein ligand 1; SD, standard deviation.

### Primary outcome

#### Prevalence of PD-L1 expression

Based on test results from the central laboratory, the prevalence of high PD-L1 expression was found to be 52.4% (95% CI 45.7–59.0%; Table 2).

**Table 2:** PD-L1 expression status based on central laboratory testing results.

Variables	Total
	(N = 229)
	n (%)
Overall PD-L1 status	229
High PD-L1 (+ TC or + IC)	120 (52.4)
Low PD-L1 (− TC and − IC)	109 (47.6)
TC overall evaluation	229
+ TC (≥ 25%)	59 (25.8)
− TC (< 25%)	170 (74.2)
IC overall evaluation (% IC present = 1%)	5
+ IC (= 100%)	0
− IC (< 100% or = 0%)	5 (100)
IC overall evaluation (% IC present ≠ 1%)	224
+ IC (≥ 25%)	82 (36.6)
− IC (< 25%)	142 (63.4)

IC, Immune cells; N, number of subjects in the analysis population; n, number of subjects in the specified category; PD-1, programmed cell death protein 1, PD-L-1, programmed cell death protein death ligand 1, SD, standard deviation, TC, tumor cells.

### Secondary outcomes

#### PD-L1 expression in TC and IC

The analysis of patients in the central laboratory revealed high PD-L1 expression in TC of 59 (25.8%) patients and in IC of 82 (36.6%) patients (Table 2).

#### Concordance rates of PD-L1 testing between laboratories

A total of 46 patients with high PD-LI expression in both central and hospital laboratory tests were included for the evaluation of concordance rate. A high concordance rate of 80.4% was observed for PD-L1 expression levels between the central and hospital laboratories (kappa = 0.57; Table 3).

**Table 3:** PD-L1 testing concordance rates between laboratories.

	High PD-L1 expression status	Central laboratory result		
		Yes (%)	No (%)	Total
Hospital laboratory	Yes (%)	10 (21.7)	0	10 (21.7)
	No (%)	9 (19.6)	27 (58.7)	36 (78.3)
	Total	19 (41.3)	27 (58.7)	46
Concordance (%)	80.4			
Kappa coefficient	0.57			

N, Number of subjects in the analysis population; n, number of subjects in the specified category.

#### Initial treatment pattern for MIUBC in routine clinical practice

Overall, 50 (21.8%) patients received MIUBC therapy (Table 4). Among them, 5 (10.0%), 11 (22.0%), 14 (28.0%), and 26 (52.0%) patients underwent neoadjuvant, adjuvant, medication therapy not combined with surgery, and intravesical chemotherapy (not mutually exclusive), respectively, whereas 185 (81.9%) patients had radical cystectomy, with/without transurethral bladder resection and 33 (14.6%) patients had transurethral bladder resection only with no partial or radical cystectomy as previous or planned surgery (Table 4).

**Table 4:** Initial treatment pattern for MIUBC patients in routine clinical practice.

Variable	Total
	(N = 229)
	n (%)
Systemic/intravesical therapy	50 (21.8)
Therapy type (not mutually exclusive)	50
Systemic therapy	29 (58.0)
Intravesical therapy	26 (52.0)
Treatment status (not mutually exclusive)	50 (21.8)
Neo-adjuvant	5 (10.0)
PD-1/PD-L1 inhibitors	2 (40.0)
Chemotherapy	5 (100.0)
Adjuvant	11 (22)
Pd-1/Pd-L1 inhibitors	7 (63.6)
Chemotherapy	9 (81.8)
Other (unknown)	1 (9.1)
Medication therapy alone (not combined with surgery)	14 (28)
PD-1/PD-L1 inhibitors	6 (42.9)
Chemotherapy	12 (85.7)
Intravesical chemotherapy	26 (52)
Epirubicin/pirarubicin	9 (34.6)
BCG vaccine	2 (7.7)
Gemcitabine	8 (30.8)
Lobaplatin	14 (53.8)
Any cystectomy or transurethral bladder resection for previous and planned surgery	226
Transurethral bladder resection only, no partial or radical cystectomy	33 (14.6)
Partial cystectomy, with/without transurethral bladder resection	5 (2.2)
Radical cystectomy, with/without transurethral bladder resection	185 (81.9)
Partial and radical cystectomy, with/without transurethral bladder resection	3 (1.3)

The initial treatment pattern is defined as the pattern of treatment at baseline visit.

N, number of subjects in the analysis population; n, number of subjects in the specified category; PD-1, programmed cell death protein 1, PD-L1, programmed cell death protein death ligand 1.

### Exploratory outcomes

#### Correlation between PD-L1 expression and demographic characteristics

The exploratory analysis was conducted by the subgroup analysis to investigate the multifactor correlation between demographic characteristics and PD-L1 expression. The results showed no significant association between age, gender, primary tumor stage, metastatic disease, regional lymph nodes, tobacco use, or AJCC stage with high PD-L1 expression (all *p* > 0.05; Table 5). No baseline characteristics were found to have a significant correlation with PD-L1 expression during the multivariate analysis (Supplementary Table S1).

**Table 5:** Prevalence of high PD-L1 expression levels by demographic subgroups.

Variables	N	Prevalence of high	*p*-value
		PD-L1 expression n (%)	
Age (years)			
< 70	121	60 (49.6)	0.367
≥ 70	108	60 (55.6)	
Gender			
Male	197	103 (52.3)	0.930
Female	32	17 (53.1)	
Primary tumor site			
T2	129	64 (49.6)	0.518
T3	58	34 (58.6)	
T4	39	20 (51.3)	
Metastatic disease			
M0	188	95 (50.5)	0.541
M1	8	5 (62.5)	
MX	30	18 (60.0)	
Tobacco use			
Ever	85	50 (58.8)	0.135
Never	144	70 (48.6)	
Regional lymph nodes			
N0	160	87 (54.4)	0.471
N1	26	10 (38.5)	
N2	21	12 (57.1)	
N3	4	1 (25.0)	
NX	15	8 (53.3)	
AJCC stage			
Stage II	117	59 (50.4)	0.418
Stage III	86	44 (51.2)	
Stage IV	23	15 (65.2)	

N, Number of subjects with nonmissing charateristics in the analysis population; n, number of subjects in the specified category; PD-1, programmed cell death protein 1, PDL-1, programmed cell death protein death ligand 1.

#### Expression of CD8+ cells and TMB

The exploratory analysis revealed that 102 (44.5%) patients had high (> median value of 3%) and 126 (55.0%) patients had low (≤ median value of 3%) expression of CD8+ T cell with a mean percentage of CD8+ cells of 6.1%. Approximately, more than half of the patients (n = 124, 54.1%) had shown high TMB, with a mean TMB of 14.14 mut/Mb (Table 6).

**Table 6:** Expression of CD8+ T cells and tumor mutation burden.

Variable	Total (n = 229)
Percentage of CD8+ T cells, %	
Mean (SD)	6.1 (8.8)
Median (Q1, Q3)	3.0 (1.0, 7.0)
Percentage of CD8+ T cells category, n (%)	
CD8+ High (> median value 3.0%)	102 (44.5)
CD8+ Low (≤ median value 3.0%)	126 (55.0)
Missing	1 (0.4)
TMB, Mut/Mb	
Mean (SD)	14.1 (11.1)
Median (Q1, Q3)	11.0 (7.2, 17.6)
TMB category, n (%)	
TMB high (> 10 mut/Mb)	124 (54.1)
Non- TMB high (≤ 10 mut/Mb)	92 (40.2)
Missing	13 (5.7)

N, Number of subjects in the analysis population; n, number of subjects in specified category; Q1, lower quartile; Q3, upper quartile; SD, standard deviation; TMB, tumor mutation burden.

#### Correlation between PD-L1 expression and percentage of CD8+ T cells and TMB

A significant positive correlation was observed between the percentage of TC with membrane PD-L1 positivity and CD8+ T cells (0.34; 95% CI, 0.22–0.45; *p* < 0.001), between the percentage of IC present and CD8+ T cells (0.76; 95% CI 0.70–0.81; *p* ˂ 0.001) and between the percentage of IC with membrane PD-L1 positivity and CD8+ T cells, (0.44; 95% CI 0.33–0.54; *p* < 0.001), respectively. Moreover, a weak but statistically significant positive correlation was observed between the percentage of IC present and TMB (0.15; 95% CI 0.01–0.27; *p* = 0.032) and between the percentage of IC with membrane PD-L1 positivity and TMB (0.16; 95% CI 0.02–0.29; *p* = 0.020) (Supplementary Table S2).

## Discussion

This prospective study described the prevalence of high PD-L1 expression in Chinese patients with MIUBC at 17 hospitals and explored the correlation between PD-L1 expression with tumor biomarkers such as TMB and CD8+ T cells. To the best of our knowledge, this is the first and largest prospective, multicenter study to evaluate the PD-L1 expression status and the association of PD-L1 expression with tumor immune biomarkers in patients with MIUBC using fresh tumor samples in China.

PD-L1/PD-1 inhibitors have demonstrated promising outcomes in selected patients with BC^25,26^. However, the benefits are limited to a small subset of patients. To predict the response to PD-L1/PD-1 inhibitors in MIBC, multiple biomarkers such as PD-L1 expression and TMB have been explored. Despite several considerations regarding the role of PD-L1 expression as a predictive biomarker, it is still considered the most extensively researched and effective biomarker in BC. The positive PD-L1 expression rate in MIBC in China can assist in determining future treatment strategies.

In our study, 52.4% of patients with MIUBC had high PD-L1 expression. This is in line with IMvorgor010 held in Europe, Asia, Australia, and North America, and PURE 01 trial conducted in Europe, reporting a prevalence of 48% to 59% for high PD-L1 expression^27,28^. In contrast to our results, only 23.5% of South Korea patients with MIUBC have shown high PD-L1 expression (which evaluates PD-L1 expression using the same assay and cutoffs as in our study^29^. One potential reason for this discrepancy could be the utilization of archival tumor samples, whereas our study utilized newly acquired tumor samples within 60 days.

Although, high expression of PD-L1 on TC represents the antitumor activity of PD-L1 inhibitors, interestingly, it has been suggested that IC have also been involved in the response pathway as many clinical trials have shown that patients with PD-L1-negative tumors also responded to ICI therapy, indicating a potential contribution of PD-L1 from host IC^30,31^. Thus, the interest of research has shifted to analyze the PD-L1 expression in both IC and TC rather than TC alone^30^. Our study showed a numerically higher levels of PD-L1 expression in IC than TC (36.6% vs 25.8%), which justifies this hypothesis. The results from our study are consistent with previous studies with respect to the percentage of IC and TC. A study by Wang et al. reported a high PD-L1 expression of 55% versus 23% in IC versus TC in patients with untreated urothelial bladder cancer receiving radical cystectomy or transurethral resection^32^. Another study by Pichler et al. reported a high PD-L1 expression of 61.4% versus 40% in IC versus TC in high-risk patients with extravesical disease and pelvic node involvement and receiving radical cystectomy without cisplatin-based adjuvant therapy^33^. The discrepancies between these percentages might be due to different locations, different cohort sizes, tumor heterogeneity, or different IHC methodologies and initial treatment patterns^32^.

Furthermore, we have also determined the concordance rate of the PD-L1 expression between central and hospital laboratories. The results showed a high concordance rate of 80.4%; however, with a kappa coefficient of 0.57, we considered the quality of concordance to be only a moderate agreement between both laboratories. This could be due to different factors that may affect the concordance, such as analytical factors, transportation or storage of samples, and quantity of samples. Furthermore, only 46 patients from central and hospital laboratories with high PD-L1 status were analyzed for concordance. As all 46 patients were tested at 17 different hospitals in China, there may be biasness between different hospital laboratories.

In the present study, 185 (81.9%) enrolled patients received radical cystectomy, whereas only 5 patients (10%) had undergone neoadjuvant chemotherapy and 11 patients (22%) had undergone adjuvant chemotherapy in our study. Neoadjuvant cisplatin-containing combination chemotherapy is a preferred regimen in the current European Association of Urology (EAU) guidelines and NCCN guidelines^34^. However, earlier studies suggested an overall survival benefit with adjuvant cisplatin-based chemotherapy for pathologic T3, T4, or N + disease at cystectomy, if it was not provided as neoadjuvant^35^. The use of neoadjuvant and adjuvant chemotherapies is low, nevertheless, in Europe or the Unites States. Earlier studies in the United States had shown that around 16.9% of patients with MIUBC received neoadjuvant chemotherapy according to the National Cancer Database^36^, whereas only 12% of patients from Western and Central Europe had undergone neoadjuvant chemotherapy^37^. Numerous factors, such as concerns about delay in receiving radical cystectomy (because of the time needed for 3–4 cycles of neoadjuvant chemotherapy or perioperative toxicity), the intolerance of elderly patients toward chemotherapy, patient refusal of high cost, more medical expenses, and confidence of surgeons in their operating skills, may appropriately influence the decision on usage of neoadjuvant chemotherapy. Despite this, our study offered the inaugural insight into the treatment paradigm of NMIBC in China, facilitating an understanding of the present circumstances and delineating potential avenues for enhancement.

The current study also investigated the multifactorial correlation between demographic/clinical characteristics and PD-L1 expression and infiltration of CD8+ T cells, showing a significant association of PD-L1 expression with gender, primary tumor stage (T2), regional lymph nodes (N0) and AJCC staging. Tobacco use and regional lymph nodes staging were also associated with the differential expression of PD-L1 in patients with MIUBC. However, the previous study by Holland et al. showed that the age and gender of the patients were not significantly associated with PD-1/PD-L1 expression^38^. Another meta-analysis also showed a nonsignificant association of high PD-L1 expression with gender^39^. Moreover, our results were also inconsistent with the earlier European studies that showed a nonsignificant association of PD-L1 expression with tumor stage and lymph node staging^40–42^. The inconsistency in these results may be due to the heterogenicity of different populations of these studies^30^.

The immune tumor microenvironment has been considered crucial to investigate the prognostic marker^8,43^. In the present study, we have observed high TMB expression in 54.1% of patients, with a mean TMB of 14.14 mut/Mb. This was similar to a recent study conducted by Kang et al. 2022 that reported a prevalence of high TMB of 38.1%, 60.3%, and 43.6% in overall bladder cancer, early stage BC, and advanced-stage BC, respectively^44^. With respect to a median TMB of 11.0 mut/Mb (interquartile range [IQR]: 7.2, 17.6 mut/Mb) in the current study, the PURE01 trial had also showed similar results with 11.4 mut/Mb (IQR: 7, 14 mut/Mb)^20^. We also observed a high expression of CD8 + T cells in 44.5% of patients with MIUBC. The correlation analysis between TC and IC with PD-L1 expression showed a weak and positive association of TC (correlation coefficient: 0.27) and IC (correlation coefficient: 0.34) with high PD-L1 expression and infiltrated CD8+ T cells. In addition, we have also observed a weak and positive correlation of PD-L1 with TMB in TC (correlation coefficient: 0.06) and in IC (correlation coefficient: 0.19). Interactions within the tumor microenvironment are complex. High TMB can contribute to tumorigenesis but may also indicate a greater number of neoantigens. These neoantigens can activate CD8+ cytotoxic T-cells, thus promoting T cell-mediated antitumor effects^45^. However, this situation may also impose selective pressure that result in the upregulation of PD-L1 expression by tumor cells as a mechanism to inhibit T lymphocyte proliferation and immune function in T cells^46^. A recent study by Chen et al. had shown that higher levels of CD8+ T cells (HR = 0.66, 95% CI = 0.49–0.90, *p* = 0.008) and TMB (HR = 0.68, 95% CI = 0.50–0.92,* p* < 0.0129) were associated with improved overall survival indicating CD8+ T cells and TMB to be independent prognostic biomarkers for predicting the survival of patients with MIUBC in response to treatment with ICI^47^. Although various clinical studies have adopted PD-L1 status for screening patients suitable for ICI treatment, the combination of PD-L1 with other predictive biomarkers, such as TMB and CD8+ T cells, might be a more feasible approach to select the right subset of patients for appropriate ICI therapy.

Although the study protocol, standard operating procedure of sample, and data collection were prospectively designed before the initiation of the study, this study has several limitations. The quantity of samples was limited, which may have led to the loss of some data during the biomarker assay. Therefore, some pre-analytic procedures of samples may have influenced the results. Our cohort was based on the Chinese population and only included the patients who were hospitalized in the study sites. In addition, a limited number of patients with PD-L1 expression status were analyzed for concordance from both hospital and central laboratories. Moreover, the study was a part of routine clinical practice that may have variations in clinical measurements.

## Methods

### Study design

This was a multicenter, prospective, epidemiologic study conducted in 17 hospitals across China to assess the prevalence of PD-L1 expression in patients with newly diagnosed MIUBC, their initial treatment patterns, and the correlation of PD-L1 expression with other biomarkers, such as CD8+ T cells and tumor mutation burden (TMB). The trial is registered with ClinicalTrials.gov. (NCT03433924).

### Patients

Eligible patients included were those aged ≥ 18 years who had histologically or cytologically confirmed MIUBC; were previously untreated with any systemic chemotherapy, radiotherapy, investigational product, biologic, or hormonal therapy for cancer; and could provide newly acquired tumor sample within 2 months (by transurethral resection, cystectomy, or biopsy) before the enrollment for PD-L1 testing. The study was planned for a sample size of N = 400; due to slower recruitment than expected, the enrollment was terminated at N = 248, which could also provide clinically accepted precision for the estimate of a high PD-L1 expression rate. Inclusion criteria for the patients involved: (i) ≥ 18 years of age at the time of screening (ii) signed informed consent form (iii) patients who have not been previously treated with any systemic chemotherapy, radiotherapy, investigational product, biologic, or hormonal therapy for cancer treatment (iv) all patients must be able to provide a newly acquired tumor sample within 60 days before enrollment by cystectomy, transurethral resection or biopsy. Exclusion criteria involved: (i) patients exposed to prior immune-mediated therapy or other anti-CTLA-4, anti-PD-1, anti-PD-L1, or anti-PD-L2 antibodies, including therapeutic anticancer vaccines (ii) patients with concurrent chemotherapy, investigational product, or biologic therapy for cancer treatment.

### Study outcomes

The primary endpoint was the prevalence of high PD-L1 expression in newly diagnosed patients with MIUBC. The secondary endpoints were different PD-L1 expression levels in tumor cells (TC) or immune cells (IC) in Chinese patients with MIUBC, the concordance rate of high PD-L1 test results between study central and each site’s laboratory, and the initial treatment pattern, such as surgery and medication before and after surgery in usual clinical practice in China. Furthermore, the exploratory analysis was conducted by a subgroup analysis to investigate any correlation among (i) demographic characteristics and the expression of PD-L1 and tumor biomarkers, such as CD8+ T cells and TMB, (ii) TC and IC with membrane PD-L1 positivity and CD8+ T cells, and (iii) TC and IC with membrane PD-L1 positivity and TMB.

### Data collection

The following data on age, gender, body mass index (BMI), nicotine use, The American Joint Committee on Cancer (AJCC) stage, tumor stage (T: Tumor size; N: lymph Nodes; M: Metastasis) and the initial treatment pattern, such as surgery, chemotherapy or radiotherapy, were collected from all patients. The treatment given was in accordance with the real-world clinical practice during the study with an uninfluenced decision of the physician. The test results for PD-L1 and other biomarkers were also collected. No experimental drug was given to patients during the study.

### Sample collection and processing for biomarkers assessment

The expression of PD-L1 and other biomarkers, including IC subset CD8+ T cells, TC, and TMB, were assessed for all the enrolled patients at baseline in the central laboratory (Teddy Clinical Research Laboratory, Shanghai, China). Freshly collected tumor samples selected for the study were sufficient in quantity for the evaluation of PD-L1 expression, which were utilized for the immunohistochemical (IHC) analysis and for preparing FFPE blocks.

Around 10 FFPE tissue sections of 4–5 µm thickness were collected from each patient for the central laboratory. Of these, 3, 2, and 5 samples were used for PD-L1, TC and IC subsets, and TMB analysis, respectively. In case of insufficient sections, the sections were collected for biomarker testing as per the priority—PD-L1 (3 sections) > CD8 (2 sections) > TMB (5 sections); however, a minimum of 3 sections were required for the analysis of PD-L1. The analysis was performed on consecutive slides from the same block of the same tissue sample.

The concordance rate of PD-L1 expression between the central and hospital laboratories was estimated using additional 3 tissue sections collected at 10 hospital laboratories.

### Assessment of PD-L1 expressions and other biomarkers at baseline

The expression of PD-L1 was estimated in TC and IC (defined as T cells and tumor-infiltrating immune cells [macrophages/dendritic cells] by IHC assay using Ventana SP263 (Ventana, Tucson, AZ). SP263 is a recombinant rabbit monoclonal antibody that binds to PD-L1 in FFPE tissue sections. Specific antibody localization was done by haptenated secondary antibodies in combination with multimeric anti-hapten-horseradish peroxidase using OptiVIEW DAB IHC Detection Kit on a VENTANA BenchMark ULTRA instrument. The specific antibody–enzyme complex was then visualized with a precipitating enzyme reaction product.

High PD-L1 expression was defined as PD-L1 expression by ≥ 25% TC or IC based on IHC. Notably, the percentage of tumor area occupied by any tumor-associated IC (IC present [ICP]) was used to determine IC expression (defined as the percentage area of ICP exhibiting PD-L1–positive IC staining). If the percentage of ICP in the tumor area was > 1%, PD-L1 expression was considered high when ≥ 25% of IC showed PD-LI expression. If the percentage of ICP in the tumor area is 1%, PD-L1 expression was considered high when 100% of the IC are stained or showed PD-LI expression. PD-L1 expression was considered low for samples that failed to meet high PD-L1 expression criteria.

The expression of CD8+ T cells was analyzed using IHC, whereas TMB was assessed using the next-generation sequencing, which was used to identify a wide range of mutations. TMB was defined as number of somatic mutations in the coding region per megabase; mut/Mb (including single nucleotide variants and small insertions and deletions). TMB ˃10 mut/Mb was considered high and ˂10 mut/Mb as low.

### Sample size determination

The previous study by Powles et al. 2017 had reported a 50% of prevalence rate of high PD-L1 expression in patients with metastatic urothelial cancer^48^. With a sample size of 400, the precision estimate (half-length of 95% confidence interval [CI]) of a high PD-L1 expression rate is expected to be in an acceptable range of 4.9–4.8%. The precision of the concordance rate is estimated to be between 6.4 and 4.2% with 200 patients assuming a concordance range of 70–90% between central and hospital laboratories.

### Statistical analysis

Descriptive statistics were used to describe continuous variables, and categorical variables were summarized using frequencies and percentages. All patients who met the study eligibility criteria and had valid data of PD-L1 expression status were included in the analyses. The concordance rate of PD-L1 expression between the central and each site’s laboratory was calculated as the number of patients with the same high PD-L1 expression status based on central and hospital laboratories divided by the total number of patients with valid test results from both central and hospital laboratories. The kappa coefficient was used for estimating the correlation of PD-L1 testing between the central and hospital laboratories. The association between PD-L1 expression, tumor biomarkers, and demographic characteristics was tested using the Chi-square test or Fisher’s exact test. The correlation between PD-L1 + TC and TMB, PD-L1 + IC and TMB, PD-L1 + TC and CD8 + T cells, and PD-L1 + IC and CD8 + T-cells was analyzed using Spearman’s rank correlation coefficient. All the analyses were performed by using SAS software v9.4.

### Ethical considerations

This study was approved by the ethics committee of the respective study sites and conducted in accordance with the Declaration of Helsinki, Good Clinical Practice of International Conference Harmonization, and the applicable legislation on non-interventional and observational studies. Patients provided written informed consent before participation.

## Conclusion

Our study has shown a high prevalence of PD-L1 expression in Chinese patients with MIUBC. The study also provided an overview of the current treatment pattern of newly diagnosed MIUBC and a positive correlation between PD-L1 expression and tumor biomarkers, such as CD8+ and TMB. The biomarker-driven selection of appropriate patient populations and treatment settings could help in maximizing the treatment benefits to the patients. With the subsequent follow-up time, the prompting effect of biomarkers on survival is warranted in the future study.

### Supplementary Information

Supplementary Information.

## Data Availability

The datasets generated during and/or analyzed during the current study are available from the corresponding author upon reasonable request.
