# Supplementary material for: PD-L1 expression and its correlation with tumor biomarkers in Chinese urothelial bladder cancer
Source: Sci Rep. 2024 Jul 20;14:16753. doi: 10.1038/s41598-024-67508-6 (PMC11271459; doi:10.1038/s41598-024-67508-6)
Supplement: Supplementary file 1 — Supplementary Information. [file 41598_2024_67508_MOESM1_ESM.docx]

**Supplementary Table S1.** Estimated impact factors of different PD-L1 expression levels

| Model | Characteristics | N | OR | 95% CI | *p*-value |
| --- | --- | --- | --- | --- | --- |
| Univariate Model | **Age (<70 vs. ≥70)** | 229 | 1.235 | (0.71, 2.14) | 0.452 |
|  | **Gender (Male vs. Female)** | 229 | 0.887 | (0.39, 2.00) | 0.774 |
|  | **Primary tumor site (T3 vs. T2)** | 226 | 1.708 | (0.43, 6.84) | 0.450 |
|  | **Primary tumor site (T4 vs. T2)** | 226 | 0.844 | (0.19, 3.70) | 0.822 |
|  | **Metastatic disease (M1 vs. M0)** | 226 | 1.010 | (0.15, 6.61) | 0.992 |
|  | **Metastatic disease (MX vs. M0)** | 226 | 1.424 | (0.60, 3.36) | 0.419 |
|  | **Tobacco use (Ever vs. Never)** | 229 | 1.799 | (0.99, 3.26) | 0.053 |
|  | **Regional lymph nodes (N1+N2+N3 vs. N0)** | 226 | 0.413 | (0.17, 1.01) | 0.053 |
|  | **Regional lymph nodes (NX vs. N0)** | 226 | 0.837 | (0.25, 2.80) | 0.772 |
|  | **AJCC Stage (Stage III vs. Stage II)** | 226 | 1.136 | (0.25, 5.12) | 0.868 |
|  | **AJCC Stage (Stage IV vs. Stage II)** | 226 | 3.230 | (0.47, 22.38) | 0.235 |
|  |  |  |  |  |  |
| Multivariate Model | **Age (<70 vs. ≥70)** | - | - | - | - |
|  | **Gender (Male vs. Female)** | - | - | - | - |
|  | **Primary tumor site (T3 vs. T2)** | - | - | - | - |
|  | **Primary tumor site (T4 vs. T2)** | - | - | - | - |
|  | **Metastatic disease (M1 vs. M0)** | - | - | - | - |
|  | **Metastatic disease (MX vs. M0)** | - | - | - | - |
|  | **Tobacco use (Ever vs. Never)** | 229 | 1.534 | (0.890, 2.642) | 0.123 |
|  | **Regional lymph nodes (N1+N2+N3 vs. N0)** | - | - | - | - |
|  | **Regional lymph nodes (NX vs. N0)** | - | - | - | - |
|  | **AJCC Stage (Stage III vs. Stage II)** | - | - | - | - |
|  | **AJCC Stage (Stage IV vs. Stage II)** | - | - | - | - |

Abbreviations-

AJCC, The American Joint Committee on Cancer; CI, confidence interval; N, number of subjects in the analysis population; OR, odds ratio

**Supplementary Table S2.** Correlation between infiltration of CD8+ T cells and PD-L1 expression.

| Related Factors | N | Spearman's rank correlation coefficient | 95% CI | *p*-value |
| --- | --- | --- | --- | --- |
| The percentage of TC with membrane PD-L1 positivity and TMB | 216 | 0.05 | (-0.08, 0.18) | 0.441 |
| The percentage IC present and TMB | 216 | 0.15 | (0.01, 0.27) | 0.032 |
| The percentage of IC with PD-L1 positivity and TMB | 216 | 0.16 | (0.02, 0.29) | 0.020 |
| The percentage of TC with membrane PD-L1 positivity and CD8+ T cell | 228 | 0.34 | (0.22, 0.45) | <0.001 |
| The percentage IC present and CD8+ T cell | 228 | 0.76 | (0.70, 0.81) | <0.001 |
| The percentage of IC with PD-L1 positivity and CD8+ T cell | 228 | 0.44 | (0.33, 0.54) | <0.001 |

Abbreviations-

IC, immune cell; N, number of subjects in the analysis population; PD-L-1, programmed cell death protein death ligand 1; TC, tumor cell; TMB, tumor mutation burden
